# Entomotoxic Activity of *Prasiola crispa* (Antarctic Algae) in *Nauphoeta cinerea* Cockroaches: Identification of Main Steroidal Compounds

**DOI:** 10.3390/md17100573

**Published:** 2019-10-10

**Authors:** Graziela Holken Lorensi, Raquel Soares Oliveira, Allan P. Leal, Ana Paula Zanatta, Carlos Gabriel Moreira de Almeida, Yuri Correia Barreto, Maria Eduarda Rosa, Patrícia de Brum Vieira, Carlos José Brito Ramos, Filipe de Carvalho Victoria, Antônio Batista Pereira, Valéria LaneuvilleTeixeira, Cháriston André Dal Belo

**Affiliations:** 1 Laboratório de Neurobiologia e Toxinologia (LANETOX),Universidade Federal do Pampa, Campus São Gabriel, São Gabriel, RS 97307-020, Brazil; grazielahl@gmail.com (G.H.L.); raquelsoaresoliveira@yahoo.com.br (R.S.O.); anapaulabiotech@gmail.com (A.P.Z.); barreto78@outlook.com (Y.C.B.); rosaedumaria@gmail.com (M.E.R.); patriciasbrum@yahoo.com.br (P.d.B.V.); 2 Instituto do Cérebro (INSCER), Pontifícia Universidade Católica do Rio Grande do Sul, Porto Alegre, RS 90610-000, Brazil; carlos.gabriel@acad.pucrs.br; 3 Núcleo de Estudos da Vegetação Antártica (NEVA), Universidade Federal do Pampa, Campus São Gabriel, São Gabriel, Rio Grande do Sul 97307-020, Brazil; filipevictoria@unipampa.edu.br (F.d.C.V.); antoniopereira@unipampa.edu.br (A.B.P.); 4 Grupo de Pesquisa em Estresse Oxidativo e Sinalização Celular, Universidade Federal do Pampa, Campus São Gabriel, São Gabriel, RS 97307-020, Brazil; 5 Programa de Pós-Graduação em Ciências e Biotecnologia, Instituto de Biologia, Universidade Federal Fluminense, Centro, Niterói, RJ 24020-141, Brazil; valerialaneuville@gmail.com; 6 Programa de Pós-Graduação em Biodiversidade Neotropical, Universidade Federal do Estado do Rio de Janeiro, Rio de Janeiro, RJ 22290-255, Brazil; cjbramos@gmail.com; 7 Programa de Pós-Graduação em Bioquímica Toxicológica, PPGBtox, Universidade Federal de Santa Maria, UFSM, Bairro Camobi, Santa Maria, RS 9705-900, Brazil; allan-leal@hotmail.com

**Keywords:** Antarctic algae, biological activity, insecticide, marine steroids, marine natural products

## Abstract

*Prasiola crispa* is a macroscopic green algae found in abundance in Antarctica ice free areas. *Prasiola crispan*-hexaneextract (HPC) induced insecticidal activity in *Nauphoeta cinerea* cockroaches after 24 h of exposure. The chemical analysis of HPC revealed the presence of the followingphytosterols: β-sitosterol, campesterol and stigmasterol. The incubation of cockroach semi-isolated heart preparations with HPC caused a significant negative chronotropic activity in the heartbeats. HPC affected the insect neuromuscular function by inducing a complete inhibition of the cockroach leg-muscle twitch tension. When the isolated phytosterols were injected at in vivo cockroach neuromuscular preparations, there was a progressive inhibition of muscle twitches on the following order of potency: β-sitosterol > campesterol > stigmasterol. HPC also provoked significant behavioral alterations, characterized by the increase or decrease of cockroach grooming activity, depending on the dose assayed. Altogether, the results presented here corroborate the insecticide potential of *Prasiola crispa* Antarctic algae. They also revealed the presence of phytosterols and the involvement of these steroidal compounds in the entomotoxic activity of the algae, potentially by modulating octopaminergic-cholinergic pathways. Further phytochemical-combined bioguided analysis of the HPC will unveil novel bioactive compounds that might be an accessory to the insecticide activity of the algae.

## 1. Introduction

Antarctica is the fifth largest continent on earth; its territory covers about 10% of our planet land surface with 14 million square kilometers [1]. Antarctica is known for its extreme climate conditions, being the coldest, driest and windiest continent on the planet [2]. Besides its permanent low temperatures, the Antarctica´s rate of precipitation is comparable to the world’s driest deserts [3]. Life on the Antarctic continent is basically composed of algae, fungi, bacteria (actino-mycetes) and some plant species [1,3]. Thus, Antarctica´s extreme conditions provide a perfect environment for the living organisms to synthetize new chemicals with a range of different biological properties, including entomotoxic molecules [4]. Among the vegetal organisms well adapted to the Antarctica´s stressful conditions, there are few algae species, including green algae from the order Prasiolales. Therefore, algal secondary chemistry is driven by its biogeography [5,6,7].

**Figure 1:**
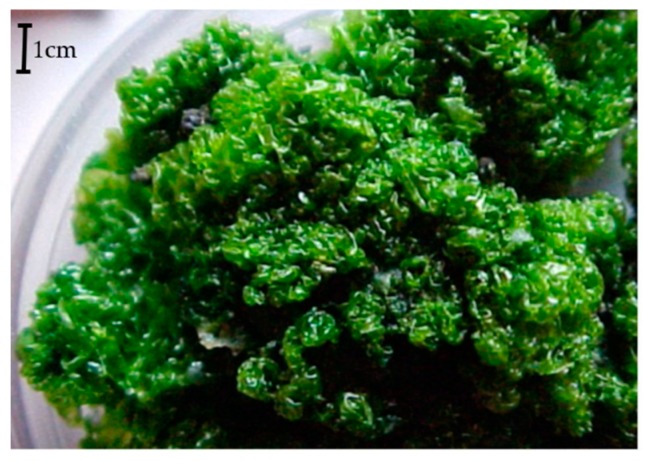
*Prasiola crispa* (Lightfoot) Meneghini collected in ice-free areas near to Arctowski Polish Base Region, Admiralty Bay, King George Island, Antarctica. Picture by Antônio Pereira Batista.

*Prasiola crispa* (Lightfoot) Meneghini (Figure 1), is a macroscopic terrestrial green algae which thalli of the pseuparenchymatic laminar form reaches 1 × 0.5 cm, forming a mass of 2.0 to 6 cm thickness, being the Antarctica´smost studied algae from the ecological perspective. This algae grows abundantly in terrestrial habitats of meltwater zones [8,9,10], and it is associated with marine bird colonies and seal aggregations areas [11]. Seaweeds, such as the Prasiolales order, contain a considerable quantity of phytosterols, however, few studies about its biological effects are found in the literature [12,13,14], especially those related to their pharmacological and toxicological potentials.

In this work, we have identified the mechanisms involved in the entomotoxic activity of *Prasiola crispa’s n*-hexane extract. To accomplish this, we have employed *N. cinerea* cockroaches, which is a practical, inexpensive, and rapid model for neurotoxicological studies [15,16,17]. The entomotoxic effect of this algae was associated to the inner presence of different phytosterols in its extract, which reinforces its biotechnological potential as a natural insecticide [18,19]. 

## 2. Results

### 2.1. Insecticidal Activity of n-Hexane Extract of Prasiola crispa

**Figure 2:**
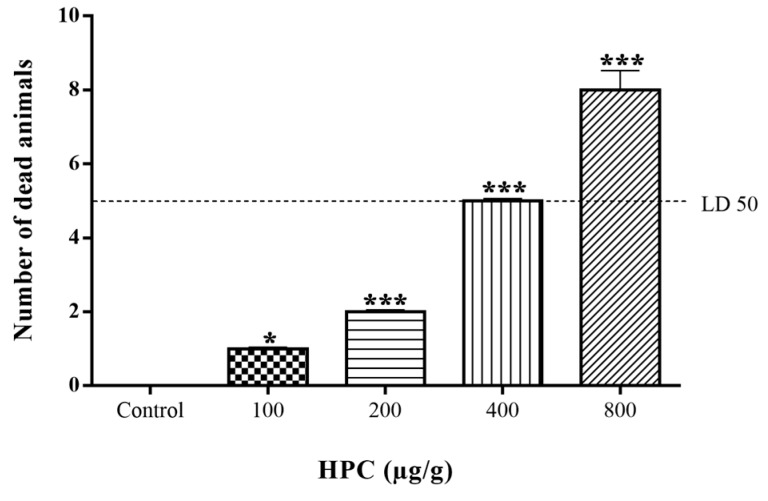
Insecticidal activity of *n*-hexane extract of *P. crispa* (HPC) on *N. cinerea* cockroaches. The results were expressed as the number of dead animals per dose. Statistical analyses were performed by one-way ANOVA followed by the Dunnett’s test. * *p* < 0.05 (*n* = 3); *** *p* < 0.001 (*n* = 3), compared to control 10% DMSO.

The insecticidalactivity of HPC was confirmed in cockroaches, by injecting four doses of the extract (100, 200, 400, and 800 μg/g of body weight). All doses of HPC induced lethality in *N. cinerea*. The dose of 400 µg/g revealed the LD_50_, killing 50% of the animals in 24 h (Figure 2). In control assays, with the incubation with 10% DMSO alone, there was no animal death.

### 2.2. The AChE Activity in Cockroach Brain Homogenates Was not Affected by HPC

The evaluation of *N. cinerea* brain homogenates AChE activity, before and after treatment with different doses of HPC (100, 200, 400 and 800 µg/g of body weight), showed no alterations, when compared to the control (10% DMSO). Data not shown.

### 2.3. Effects of HPC on the Cockroach’s Heart Rate

**Figure 3:**
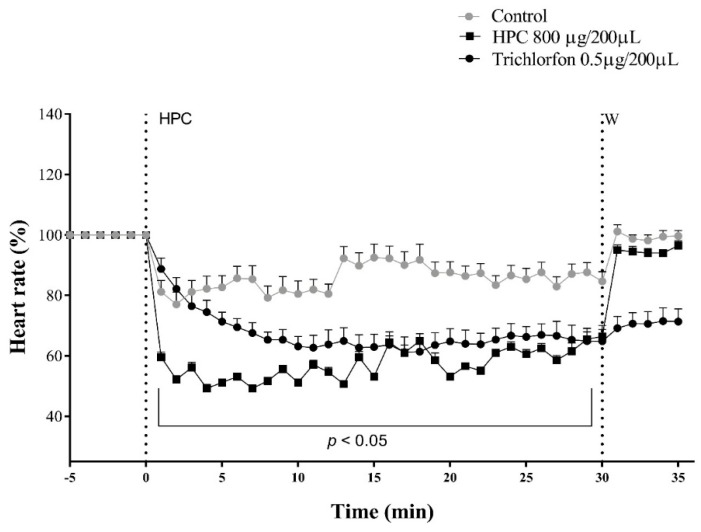
Cardiotoxic effect of HPC on*N. cinerea* cockroaches. The recordings were made during 30 min. Statistical analyses were performed using two-way ANOVA followed by Bonferroni’s test compared to control. *p* < 0.05 (*n* = 9); Control: 10% DMSO in insect saline.

The assay of HPC (100–800 µg/200 µL), on cockroach semi-isolated heart preparation induced a significative decrease of heartbeats, only with the maximum concentration (Figure 3). On this set of protocols, the incubation of the preparation with 10% DMSO, induced only a slight decrease in the heart frequency. However, when HPC 800 μg/200 µL was added to the preparation, there was a negative chronotropic modulation of heart rates, with a final inhibition of about 50%, in 30 min recordings (*n* = 9, *p* < 0.05) (Figure 3). The washout of the preparation with insect saline, reversed the HPC inhibitory activity, leading the rates to control levels. The application of Trichlorfon (Tn) 0.5 μg/200 μL, as a positive cholinergic control, inhibited the cardiac rhythm similarly to HPC, although irreversibly (Figure 3).

### 2.4. Effects of HPC on Cockroach Grooming Activity

**Figure 4:**
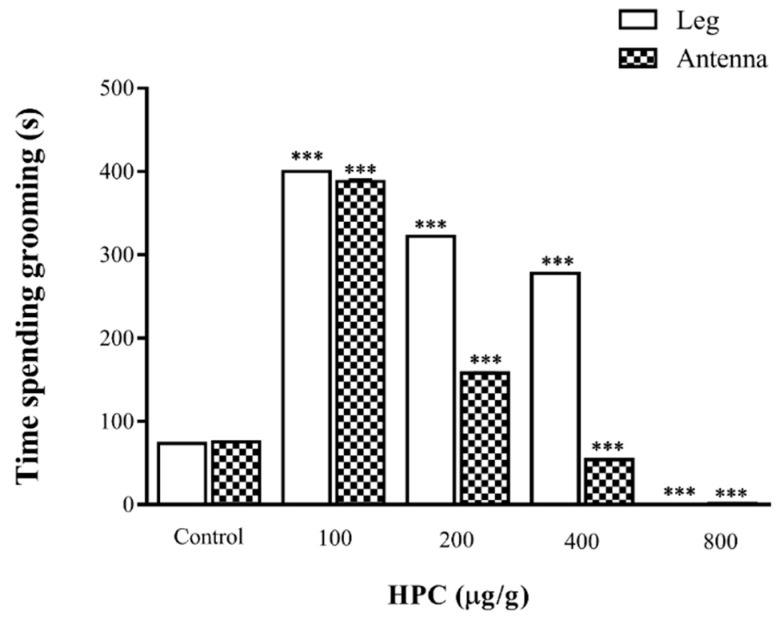
Effect of HPC on grooming behavior of *N. cinerea* cockroaches. The grooming activity was recorded during 30 min and the results were expressed as mean ± S.E.M. of the total time of grooming (in seconds). Notice the increase of grooming activity in lower HPC doses, followed by a decrease in the activity with the highest dose. The data were analyzed by one-way ANOVA followed by the Dunnett’s test compared to control. *** *p* < 0.001 (*n* = 30).

In 10% DMSO-injected cockroaches, the mean time of continuous grooming was 73.2 ± 1.2 s/30 min for legs and 75.2 ± 1.2 s/30 min for antennae (*n* = 30, respectively). Overall, the injection of HPC (100, 200, 400 and 800 µg/g body weight) on *N. cinerea* significantly affected the animals’ grooming activity. At lower concentrations, HPC (100 and 200 μg/g) induced a significative increase in the grooming activity of legs and antennae (*n* = 30, *p* < 0.001). For 100 μg/g, the increment of grooming activity of legs and antennae were 400 ± 0.7 s/30 min and 388 ± 2.8 s/30 min, respectively. At 200 μg/g, the values for legs and antennae’s grooming were 322 ± 0.7 s/30 min and 157.8 ± 1.8 s/30 min, respectively (Figure 4). At the highest dose, a complete inhibition was observed on grooming activity of legs and antennae of cockroaches (Figure 4). The injection of HPC, at 400 μg/g, induced a significant decrease on grooming activity of antennae (53.9 ± 1.0 s/30 min; *n* = 30). On the other hand, at the same concentration, HPC increased significantly the grooming of legs (277.6 ± 0.6 s/30 min; *n* = 30).

### 2.5. Neuromuscular Blockade Induced by HPC in N. cinerea Cockroaches

**Figure 5:**
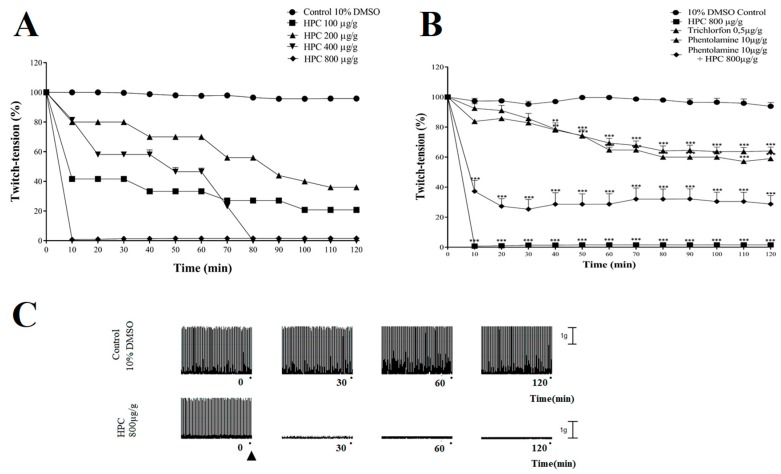
Neuromuscular blocking activity of HPC on *N. cinerea* cockroaches. The graph represents the mean ± S.E.M of six experiments (**A**,**B**). On (**A**), the neuromuscular blockade induced by different doses of HPC. On (**B**), notice the prevention of the neuromuscular blockade induced by HPC 800 µg/g body weight, by the previous incubation of phentolamine 10 µg/g in the preparation. On (**B**), the incubation of Tn 0.5 µg/g induced a similar inhibition compared to HPC in 120 min recordings. On (**C**), representative traces of the insect neuromuscular recordings in control 10% DMSO condition and during the onset of HPC (800 μg/g) activity. Statistical analyses were performed by two-way ANOVA followed by Bonferroni’s test compared to control. Control: 10% DMSO in insect saline; ▲: HPC 800 µg/g; Tn: trichlorfon; ** *p* < 0.01; *** *p* < 0.001 (*n* = 6).

In control conditions, using only 10% DMSO, there was no alteration in the muscle twitch tension of *N. cinerea*, in 120 min recordings (*n* = 6) (Figure 5A). Overall, the injection of HPC (100, 200, 400 and 800 µg/g body weight) induced a dose- and time-dependent neuromuscular inhibition in 120 min recordings (*n* = 6) (Figure 5A). When 100 and 200 μg/g HPC were assayed, there were 80 ± 0.8% and 65 ± 1.4% maximal blockages, respectively, in 120 min recordings (*n* = 6) (Figure 5A). For HPC 400 μg/g, the inhibitory effect started after 10 min, and was progressive until a complete inhibition being reached after 80 min (*n* = 6) (Figure 5A). At 800 μg/g, HPC produced a fast and progressive neuromuscular blockage, which was complete at 10 min (Figure 5A). In this set of protocols, the injection of the organophosphate compound Tn 0.5 µg/g body weight, induced 44 ± 4% blockage, in 120 min recordings (*n* = 6) (Figure 5B). To investigate the influence of octopaminergic receptors in the neurotoxic activity of HPC, we assayed phentolamine 10 μg/g body weight, a selective inhibitor of octopamine receptors. When phentolamine 10 μg/g was assayed alone, there was a 40 ± 0.6% maximum blockade of the twitch tension, in 120 min recordings (Figure 5B). The injection of phentolamine 10 μg/g previously to HPC 800 μg/g, prevented by ~70% the HPC 800 μg/g inhibitory activity (Figure 5B). Neuromuscular representative timeline traces of muscle twitch tension recordings are shown in Figure 5C.

### 2.6. Phytosterols Identified in the Prasiola crispa n-Hexane Extract

**Figure 6:**
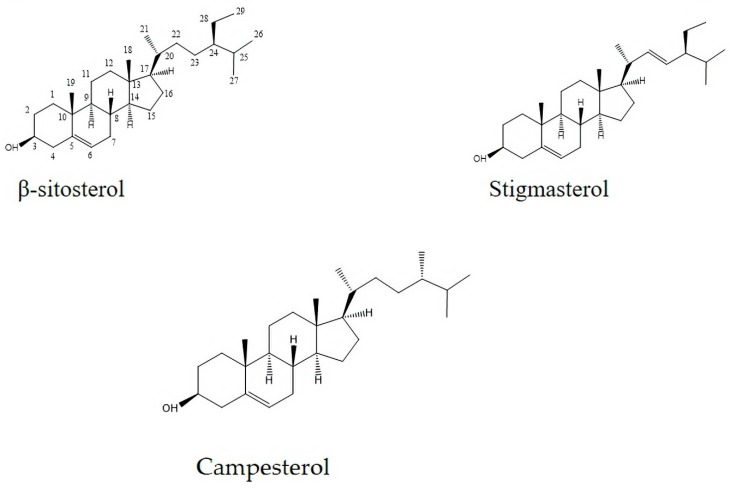
Major phytosterols identified in the *Prasiola crispan*-Hexane extract (δ in ppm, J in Hz).

The most abundant steroidal componentspresented in the extract (Figure 6). Campesterol, β-sitosterol, and stigmasterol (about 10–20% of the extract), were identified based on comparisons with standards (Sigma/Aldrich Products) and/or literature [20] (Table 1).

### 2.7. Effects of Phytosterols on Nerve-Muscle Preparation of Cockroaches

**Figure 7:**
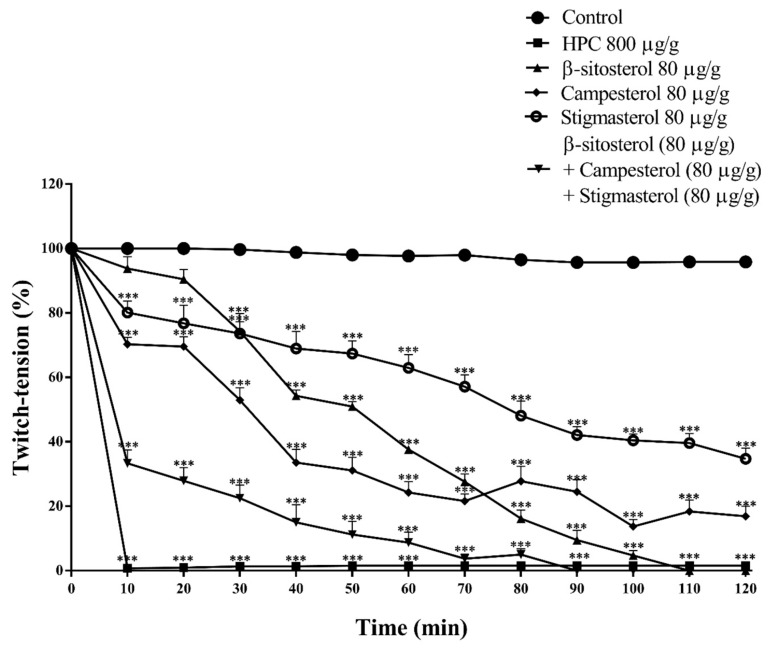
Neuromuscular blocking activities induced by β-sitosterol, campesterol, and stigmasterol in *N. cinerea* cockroaches. The results are expressed as mean ± S.E.M. Statistical analyses were performed by two-way Anova followed by Bonferroni’s test compared to control. *** *p* < 0.001 (*n* = 6).

The main compounds identified in the *P. crispa* extract were β-sitosterol, campesterol, and stigmasterol [20], which were assayed in the cockroach nerve-muscle preparations for comparison against the whole extract. Overall, the assay of the phytosterols isolated, at 80 μg/g body weight, resulted in a mimicking of the neuromuscular blockade induced by the algae whole n-hexane extract. When β-sitosterol was incubated, there was 45 ± 1.6% inhibition of the muscle twitchesat 40 min and a complete blockade in 120 min recordings (*n* = 6) (Figure 7). The analysis of campesterol and stigmasterol resulted in 80 ± 2.8% and 65 ± 3.0% neuromuscular inhibition in 120 min, respectively (Figure 7). In this set of protocols, when all the phytosterols were gathered and assayed in combination, at 80 μg/g body weight each there was an improvement of the blocking activities, resulting in a complete inhibition of the muscle twitches at 90 min (Figure 7).

## 3. Discussion

In this study we demonstrated part of the mechanisms involvedin the entomotoxic activity of *P. crispan*-hexane extract. In addition, we identified the main chemical constituents involved in *Prasiola crispa* toxic activity to insects, as being the following phytosterols: β-sitosterol, campesterol, and stigmasterol. The results obtained in different neurophysiological systems of *N. cinerea* cockroaches are discussed in detail therein, and reinforces the potential of this Antarctic alga as a natural insecticide.

In our experimental conditions, the exposure of cockroaches to HPC unveiled its insecticidal activity, agreeing with previous studies of our group using *Drosophila melanogaster* and *N. cinerea* [13]. Physiologically, the lethality induced by a natural or synthetic insecticide is a result of a chemical interaction with one of the five biological systems of the insect, which comprises, the nervous system, the production of energy, the production of cuticle, the endocrine system and water balance [21]. In the case of the nervous system, the most common target for insecticides, frequently, but not exclusively, is the insect nerve acetylcholinesterase (AChE). Under our experimental conditions, HPC displayed no activity over the brain AChE of *N. cinerea* cockroaches. Although, HPC did not alter the cockroach’s brain homogenates AChE activity, the identified phytosterols, β-sitosterol, campesterol, and stigmasterol are known to modulate this enzyme in different extents [22,23,24]. Phytosterols, are a subgroup of steroids having close structure resemblance with cholesterol and are widely distributed in herbs, fungi and animals [25]. Cholesterol, is the major constituent of eukaryotic cellular membranes, acting as secondary messenger, regulating cell signaling and physiological processes [23]. At this point, we cannot infer about the precise mechanism involved in the HPC-phytosterol-induced neurotoxicity in *N. cinerea* cockroaches. However, in some insect species, including cockroaches, cholesterolcan affect hormones and development [26]. Therefore, the absence of HPC activity over the insect AChE may be explained by the peculiar modulation that phytosterols exert against this enzyme. For example, while campesterol exhibits good anti-cholinesterase activity, stigmasterol and its correlate β-stigmasterol show to have weak bonding with AChE proteins in molecular docking analysis [27]. Taken together, the insecticide activity of HPC on cockroaches may not rely exclusively on an anti-AChE activity per se, but may be a result of the direct interaction of its chemical compounds at different targets in the insect neurophysiological system.

HPC induced cardiotoxicity in *N. cinerea*, particularly the negative chronotropic activity in semi-isolate heart preparations. This inhibitory activity of HPC resembles those induced by the cholinergic agent Tn and the *Rhinella icterica* toad poison [16]. In this regard, the insect heart receives innervation from several sources, especially from cholinergic [28] and octopaminergic nerves [29]. Octopamine (OCT) is the main cardio-modulatory neurohormone in insects and induce biphasic effects at cardiac rhythm [29,30]. Similar to other biogenic amines, octopamine signaling is mediated through binding to distinct receptors that belong to a family of metabotropic G protein-coupled receptors (GPCRs) [31]. The excitatory activity of octopamine on insect heart is related to the octopaminergic receptor subtype 2 (octopamine2) [32]. On the other hand, octopamine 1 receptors evokes the inhibition of cardiac rhythmic in insects [33,34]. Acetylcholine and AChE inhibitors also affect the heart rate in insects, inducing an increase or decrease of heart-beats, depending on the concentration [35,36,37]. Recently, our group have demonstrated the striking interaction between cholinergic-octopaminergic neurotransmission in *N. cinerea* [38]. We cannot rule out about the exact mechanism involved in the negative chronotropic activity induced by HPC, in *N. cinerea* hearts. However, since phytosterols are proven to be cholinergic agents, the activation of a cholinergic-octopaminergic neurotransmission by HPC at the insect heart levels cannot be disregarded.

In addition, HPC caused profound alterations on *N. cinerea* behavior, as demonstrated by the significant modulation of grooming activity. Despite the neural center involved in grooming activity is not completely identified, it is well established that monoamines, such as dopamine and octopamine, participate in the orchestration of this behavior, in insects [39]. In our experimental conditions, the increase of leg grooming activity rather than the antennae, in HPC-treated cockroaches, suggests a direct involvement of the neurotransmitter octopamine [40,41]. This result agreed with the inhibitory activity of HPC on heart frequency [38] and demonstrates the influence of the neurotransmitter octopamine on the deregulation of *N. cinerea* physiological and behavioral functions, in the presence of the Antarctic algae extract.

HPC also induced a severe impairment of neuromuscular function of *N. cinerea*. The inhibitory activity caused by HPC on cockroach neuromuscular function was also mimicked by the isolated phytosterols (β-sitosterol, campesterol, and stigmasterol). In insects, glutamate is an excitatory neuromuscular transmitter and γ-aminobutyric acid (GABA) is the inhibitory one [42]. It is worth of notice that, in our experimental cockroach model, the neuromuscular twitches were evoked by stimulating a cholinergic nerve, named nerve 5 (Watson, 1986). Along nerve 5, the activity is conducted centrally (coming from afferent signals of sensila) or peripherally, toward the methatoracic ganglion through monosynapticaly connected motorneurons via cholinergic [43] and octopaminergic synapsis [31]. The demonstration that phentolamine prevents the HPC neuromuscular blocking activity, suggests that an octopaminergic pathway must be involved in the algae extract peripheral toxicity. Besides, octopamine also may participate in the modulation of neuromuscular junction induced by HPC. The role of this monoamine in insects as a neurotransmitter, neurohormone, and neuromodulator is well established [44,45]. Octopamine is identified in a class of neurons of the metathoracic, abdominal, and subesophageal ganglia, which have been termed dorsal unpaired median (DUM) neurons [46,47]. The release of octopamine from dorsal unpaired neurons-DUM also coordinate the insect neuromuscular transmission, muscle contraction and direct influences other peripheral organs [48].

In addition, the evidence that the organophosphate Tn inhibits the cockroach neuromuscular junctions, similarly to other cholinergic compounds [17], corroborates the striking cooperation between octopaminergic and cholinergic pathways in the HPC entomotoxic activity at *N. cinerea*. Thus, we suggest that, in our experimental conditions, the acetylcholine-octopamine relationship would be involved in a direct modulation of the insect neuromuscular transmission, by altering the glutamatergic and GABAergic synapsis. Ultimately, this interaction of HPC with cockroach peripheral nervous system would cause lethargy of the animals, and is crucial for the insecticidal activity of HPC. Thus, the indirect effects of the isolated phytosterols at the insect neuromuscular junctions would follow the same mechanisms of organophosphate agents. Secondarily, their steroidal activities would directly reinforce the octopaminergic synapsis, improving the neuromuscular blocking activity. Further support for this hypothesis is the scientific observation that in membranes, cholesterol significantly modulates the stability, ligand-binding properties and function of several GPCRs [49]. In addition, since OCT release and reuptake systems are commanded by GPCRs in insects and are considered to be important targets for novel insecticides [31], is not far from mind to expect a direct interaction of HPC phytosterols at OCT receptors to improve its insecticidal activity.

Altogether, the results presented here corroborate the entomotoxic potential of *Prasiola crispa* Antarctic algae. They also revealed the presence of phytosterols and the involvement of these chemical compounds in the algae toxic activity in insects, potentially by an octopaminergic-cholinergic interaction. Further phytochemical-combined with a bioguided analysis of the HPC will unveil novel bioative compounds that might be accessory for the insecticide activity of the algae.

## 4. Material and Methods

### 4.1. Experimental Animals 

All experiments were performed on both sexes of adult *Nauphoeta cinerea* cockroaches (3–4 months after an adult molt). The animals were reared in laboratory conditions of controlled temperature (22–25°C) and photoperiod 12:12 L:D. All cockroaches were provided with water and dog chow ad libitum.

### 4.2. Reagents and Solutions

Unless otherwise stated, all reagents were purchased from Sigma-Aldrich, Merck, Roche, Life Technologies or BioRad. Test-solutions were prepared daily by dilution in insect saline [50], immediately before use. The insect saline is a carbonate-buffered solution freshly prepared with the following composition in mM: NaCl, 214; KCl, 3.1; CaCl_2_, 9; sucrose, 50; HEPES buffer, 5 and pH 7.2. To the biological assays with the n-hexane extract of *P. crispa* (HPC), a solution of 10% DMSO (*v*/*v*) to insect saline was used for dissolving the compound immediately before tests. All drugs were injected into the third abdominal hemocoel segment of the cockroaches at a final volume of 20 μL using a Hamilton syringe.

### 4.3. Algae Material

*Prasiola crispa* (Lightfoot) Meneghini was collected in ice-free areas Punta Ulmann, King George Island (62°04’55.6”S, 58°21’10.3”W), Antarctica. Dried vouchers on herbarium sheets are deposited at the SPF Herbarium in São Paulo, Brazil voucher (SPF428304). Seaweeds were dried in a dark chamber with air circulation at 40 °C and stored in dark plastic bagsin a freezer (−20°C). Additional screening, including washout with distilled water was performed for removal of waste and other materials before further phytochemical assays.

### 4.4. Extract Preparation 

Dried seaweeds were powdered in knife grinder (Marconi, Model MA-680, Piracicaba, SP, Brazil) and extracted with different solvents including methanol, dichloromethane, ethyl acetate, n-hexane or acetone (4 × 1.0 L), respectively, at room temperature. The resulting extracts were evaporated under reduced pressure and stored at −4°C, until biological experiments or chemical analysis.A preliminary insecticide assay was carried out with *N. cinerea* cockroaches, using all the different extracts. After that, theHPC extract was chosen because of its best potency in terms of insecticidal activity.

### 4.5. Assay for Insecticidal Activity

The insecticidal activity of HPC against *N. cinerea* was carried out according to Kagabu et al. [51]. Different concentrations of HPC (100, 200, 400, and 800 μg/g of animal) and 10% DMSO in insect saline were injected between the third and the fourth abdominal segments of cockroaches. All experiments were made in triplicate; for each dose assayed, ten cockroaches were used. The animals were kept at controlled temperature conditions (22–25°C), with water and food ad libitum, and the survival rates recorded after 24 h exposure to the test compounds.

### 4.6. Assay for Insect Acetylcholinesterase Activity

The in vitro activity of AChE was evaluated according to Stürmer et al. [50]. Six animals were treated with different doses of HPC (100, 200, 400 and 800µg/g body weight). The amount of protein in the samples was measured according to the method of [52]. The results were expressed as percentage of milliunits of AChE per milligram of protein (mU/mg protein). One milliunit of AChE was defined as the amount of enzyme.

### 4.7. Semi-Isolated Cockroach Heart Preparation

The semi-isolated cockroach heart bioassay was mounted according to Carrazoni et al. [38]. Heart beat frequencies were monitored during 30 min under a stereoscopic microscope (Olympus, Damstat, Germany). Nine cockroaches were used for each group, for each different treatment.

### 4.8. Grooming Activity

The grooming behavior of cockroaches was evaluated as previous described [41,50]. Different concentrations of HPC (100, 200, 400 and 800 µg/g of animal) or 10% DMSO in insect saline were used. The assays were carried out 2–8 h after the beginning of the light cycle and in controlled conditions of temperature (22–25 °C).

### 4.9. In Vivo Cockroach Metathoracic Coxal-Adductor Nerve-Muscle Preparation

The analysis of cockroach neuromuscular function was mounted essentially as described elsewhere [53]. Twitch-tensions were recorded, digitized and retrieved using a computer-based software AQCAD (AVS Instruments, São Carlos, SP, Brazil). Data were further analyzed using the software ANCAD (AVS Instruments, São Carlos, SP, Brazil). The data were expressed as a percentage of the neuromuscular twitch tension, before treatments.

### 4.10. Chemical Analysis

An aliquot of *Prasiola crispa n*-hexane extract (10 mg) was analyzed by NMR spectra (proton and carbon) on Varian-Unity Plus 300 MHz spectrometer, employing CDCl_3_ (99.8%, Merck, Whitehouse Station, NJ, USA), as a solventand TMS as internal standard. Chemical shifts were reported inδ(ppm) and coupling constants (J), in Hz. Multiplicities of ^13^C signals were obtained by APT. All solvents were of HPLC grade for extraction, fractionation and isolation of the sterols.TLC separations were carried out on Merck silica gel 60F-254 (0.2 mm) percolated aluminum plates. Once developed, the plates were visualized by spraying them with 2% ceric sulfate in sulfuric acid, followed by gentle heating. Fractionation was monitored by thin layer chromatography. Silica gel60 (Merck, 70–230 and 230–400 mesh), was used for column chromatography. 

The hexanic extract was fractionated by column chromatographies on silica gel Merck 70–230 and 230–400 mesh, using hexane/CH_2_Cl_2_/ethyl acetate as eluent (F_1_–F_30_). All fractions were abundant in sterols. The most abundant steroidal components were detected by NMR spectra (campesterol, β-sitosterol, and stigmasterol). The identification was based on NMR comparisons with standards (Sigma/Aldrich Products) and/or literature [54] (Table 1).

### 4.11. Statistical Analysis

Results were expressed as the mean ± SEM. Statistical analysis wereconducted by One-way ANOVA followed by Dunnet (when more than two groups were analyzed) or Two-way ANOVA followed by Bonferroni (when more than two variables were analyzed) as post hoc. Statistics and graphs were made using the Software Graphpad Prism 6.0 (Software Inc., San Diego, CA, USA).

## 5. Conclusions

This study confirmed the biotechnological potential of the Antarctic algae *P. crispa* as a natural insecticide. The mechanism of entomotoxic activity involves the presence of phytosterols in the algae. The effects of HPC in both central and peripheral nervous system of *N. cinerea* potentially involves an octopaminergic-cholinergic interaction, that induce profound alterations in the insect behavior. The importance of octopamine to insect survival and the possible deregulation of this neurotransmission for the inner steroidal molecules present in the extract reinforces the importance of phytosterols in the insecticide activity of *Prasiola crispan*-hexane extract. Further phytochemical- combined bioguided analysis of the HPC will unveil novel bioative compounds that might be accessory to the insecticidal activity of the algae.

## Figures and Tables

**Table 1:** Selected 13C- and 1H data (300 MHz in CDCl3) of the most abundant sterols found in *Prasiola crispan*-hexane extract.

C/H		Campesterol		β-sitosterol		Stigmasterol
**C3**	71.82	3.66 (1H, m)	71.71	3.66 (1H, m)	71.29	3.66 (1H, m)
**C6**	122.45	5.33 (1H;d;1.2)	119.80	5.35 (1H, s)	121.60	5.37 (1H;d; 5.33)
**C18**	11.96	0.68 (3H, s)	11.94	0.68 (3H, s)	11.21	0.70 (1H, s)
**C19**	19.21	1.00 (3H, s)	18.28	1.00 (3H, s)	20.80	1.01 (3H, s)
**C21**	18.61	0.90 (3H;d;6.5)	18.83	0.92 (3H;d; 1.5)	21.06	1.02 (3H, s)
**C22**					138.13	5.16 (1H;dd;15.0; 8.09)
**C23**					129.21	5.16 (1H;dd;14.4; 8.09)
**C26**	19.61	0.85 (3H;d;2.2)	19.48	0.80 (3H;d; 3.7)	20.40	0.80 (3H;d; 3.7)
**C27**	18.56	0.79 (3H;d;3.6)	20.40	0.84 (3H;d;2.4)	19.48	0.84 (3H;d; 2.4)
**C28**	14.00	0.77 (3H;d;3.7)				
**C29**			12.30	0.85 (3H;d; 2.2)	12.20	0.80 (3H;d;3.7)
